# A Case Report on Alpha-Fetoprotein-Positive Colorectal Cancer

**DOI:** 10.7759/cureus.53599

**Published:** 2024-02-05

**Authors:** Wendy Hui Li Yap, Hajah Maisarah Haji Awang Sharif, Amy Thien

**Affiliations:** 1 Department of General Surgery, Raja Isteri Pengiran Anak Saleha (RIPAS) Hospital, BSB, BRN; 2 Department of Pathology, Raja Isteri Pengiran Anak Saleha (RIPAS) Hospital, BSB, BRN

**Keywords:** hepatitis b(hbv), risk surveillance, hepatocellular carcinoma (hcc), colorectal cancer, afp

## Abstract

Alpha-fetoprotein (AFP) is commonly produced by hepatocellular carcinoma and yolk sac tumors, while AFP in colorectal cancer (CRC) is a rare association. We report a case of a patient with primary AFP-producing CRC, which was successfully treated with surgery and adjuvant chemotherapy. This case highlighted the importance of recognizing a case of AFP-producing CRC.

This case report discussed a 59-year-old male who had a history of hepatitis B infection, with two months of intermittent fresh per rectal bleeding. Given his previous burden of hepatitis B infection, and the serum AFP level on admission was high (212.6 ng/mL), this raised suspicions of possible hepatocellular carcinoma. Therefore, a triphasic computed tomography of the liver was performed, which revealed an incidental hepatic flexure lesion with no involvement of the liver. Subsequent colonoscopy revealed a large friable tumor obstructing the whole lumen of the proximal transverse colon. He then underwent an emergency extended right hemicolectomy. Histopathological examination showed a Duke C mucinous adenocarcinoma (T3N2b), with a satisfactory resected margin. Immunohistochemical analysis indicated that the tumor exhibited positivity for MLH1/MSH2/MSH6/PMS2 (+++) and human epidermal growth factor receptor 2 (HER2), and notably, it also stained positive for AFP. The postoperative period was uneventful, and serum AFP level eventually normalized. The patient completed eight cycles (four months) of adjuvant chemotherapy with capecitabine and oxaliplatin (CAPOX) regimen. A follow-up CT scan and colonoscopy showed no evidence of local or distant recurrence after 12 months of surveillance.

AFP may be useful for not only hepatocellular carcinoma but also CRC. In particular, this case report has fully demonstrated the unexpected incidence and emphasized the importance of early recognition and appropriate treatment to prevent potential oversights in the diagnosis of CRC.

## Introduction

The incidence of colorectal cancer (CRC) is steadily rising at an alarming rate over the decades globally. It ranks as the third most common cancer in men and the second most common in women (10.6% vs. 9.4%), according to the GLOBOCAN 2020 estimates [1]. To no surprise, Brunei Darussalam also recorded first in incidence and second in mortality for CRC, with a lifetime risk of 5%, higher than the other member states of the Association of Southeast Asian Nations (ASEAN) [2].

Alpha-fetoprotein (AFP) is rarely associated with CRC. It has been more commonly reported in the East Asia region such as Japan, Taiwan, and China but has not been described in Brunei Darussalam. AFP is often produced by hepatocellular carcinoma (HCC) and yolk sac tumors. It can be a reliable tumor marker and is used in diagnosing embryonal testicular tumors, HCC, and yolk sac tumors. It is extremely rare to have a primary AFP-producing CRC. Theories have suggested that AFP-producing tumors tend to originate from the foregut, unlike colorectal tissues, which originate from the hindgut endoderm [3]. A case-control study showed that the AFP-producing CRC has worse disease-free survival (DFS) at 23.3 months versus 26.0 months in AFP-negative CRC patients (*P *= 0.003) [4]. Here, we report a case of a patient with primary AFP-producing CRC, which was successfully treated with surgery and adjuvant chemotherapy.

## Case presentation

A 59-year-old male presented to the emergency department of RIPAS Hospital, Brunei, with four episodes of fresh per rectal bleeding. He had experienced the symptoms for two months, which had worsened with increased frequency and the passage of blood clots. His past medical history included hypertension, hyperlipidemia, ischemic heart disease (on Clopidogrel), and hepatitis B.

On clinical examination, he was well and vitally stable. The abdomen was not distended, soft, and nontender, and had no palpable mass. The per-rectal examination revealed blood-stained mucus on gloves but neither a mass nor hemorrhoids were found.

Laboratory results showed hemoglobin (Hb) of 9.4 g/dL (normal range 13.5-17.9 g/dL), and his tumor markers were elevated to 9.0 for carcinoembryonic antigen (CEA normal range 0.0-5.0 ng/mL), and AFP was 212.6 (normal range 0.9-8.8 ng/mL) at presentation. Hepatitis screening confirmed previous hepatitis B infection (Table 1).

**Table 1:** Laboratory results. AFP, alpha-fetoprotein; anti-HBc, hepatitis B core antibody; anti-HBe, hepatitis B(e) antibody; CEA, carcinoembryonic antigen; Hb, hemoglobin; HBV DNA, hepatitis B virus deoxyribonucleic acid; IgM, immunoglobulin M

Parameter	Results	Reference range
Hb (g/dL)	9.4	13.5-17.9
CEA (ng/mL)	9.0	0.0-5.0
AFP (ng/mL)	212.6	0.9-8.8
Anti-HBc IgM	Nonreactive	N/A
HBV DNA (IU/mL)	771	N/A
Anti-HBc	Reactive	N/A
Anti-HBe	Reactive	N/A

**Figure 1:**
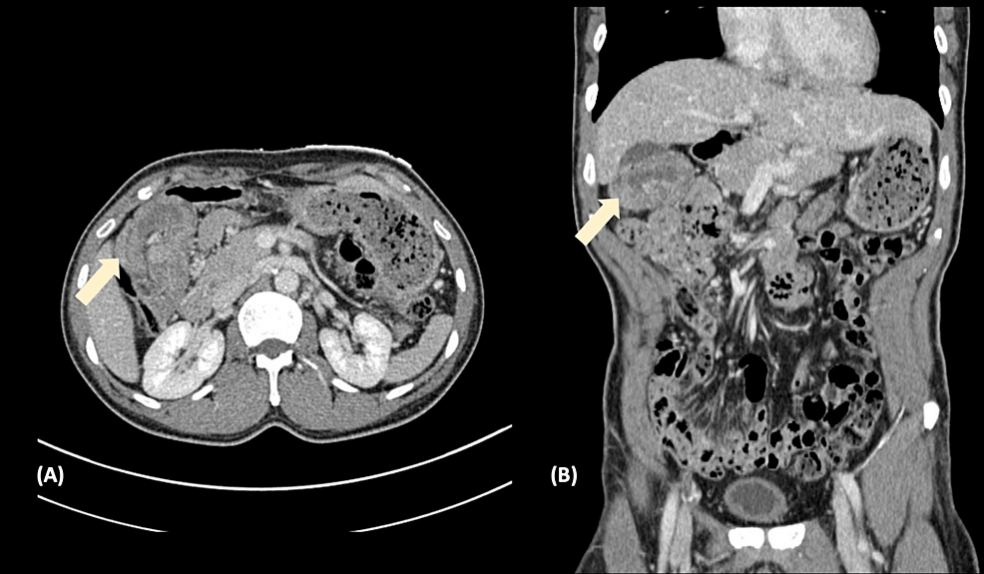
Computed tomography showing intussusception at the hepatic flexure, with the intussusceptum containing a low-attenuation mass (arrow) in the (A) axial view and (B) coronal plane.

**Figure 2:**
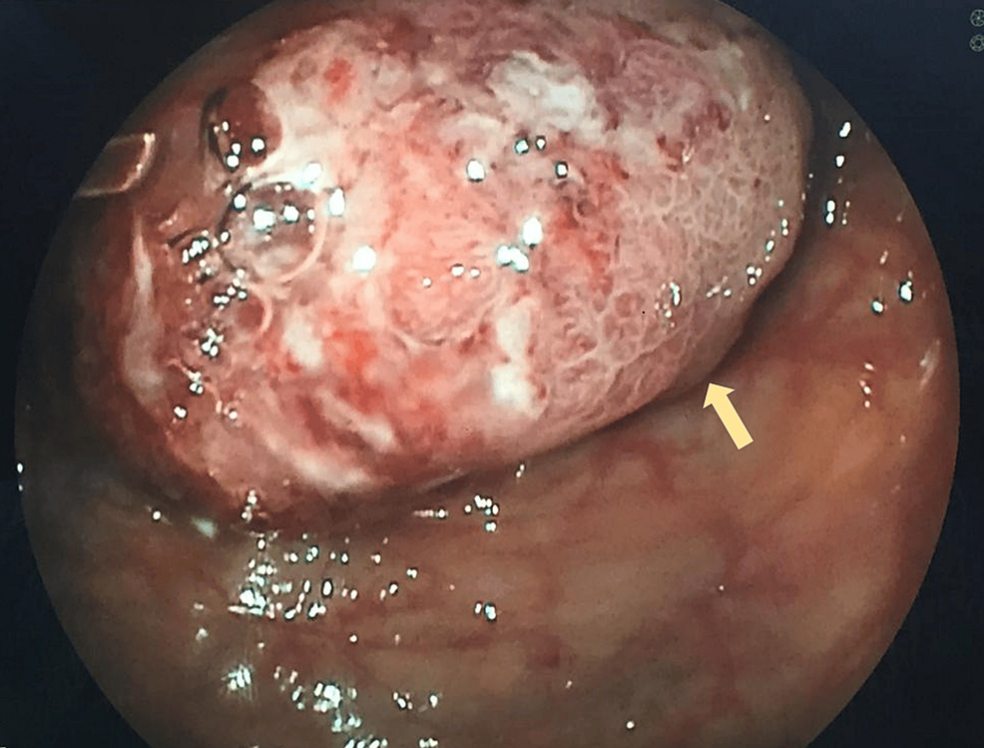
Colonoscopy showed a large friable tumor obstructing the whole of the lumen.

Ultrasound liver revealed slightly coarse parenchymal echogenicity, with a patent portal vein and no thrombosis. A triphasic computed tomography (CT) of the liver revealed no suspicious focal lesions. Instead, it showed a likely intussusception at the hepatic flexure, with the intussusceptum containing a low-attenuation mass and no evidence of bowel dilatation (Figures 1A-1B). However, the subsequent colonoscopy revealed a large friable tumor obstructing the whole lumen at the proximal transverse colon (Figure 2). Biopsy taken showed a grade 2 moderately differentiated adenocarcinoma.

The complete obstruction of the bowel lumen warranted the patient for an emergency extended right hemicolectomy with stapled functional side-to-side anastomosis. Intraoperatively, there was a tumor at the proximal transverse colon with enlarged lymph nodes along the middle colic pedicle. There was no evidence of peritoneal metastasis observed, and the liver was normal.

**Figure 3:**
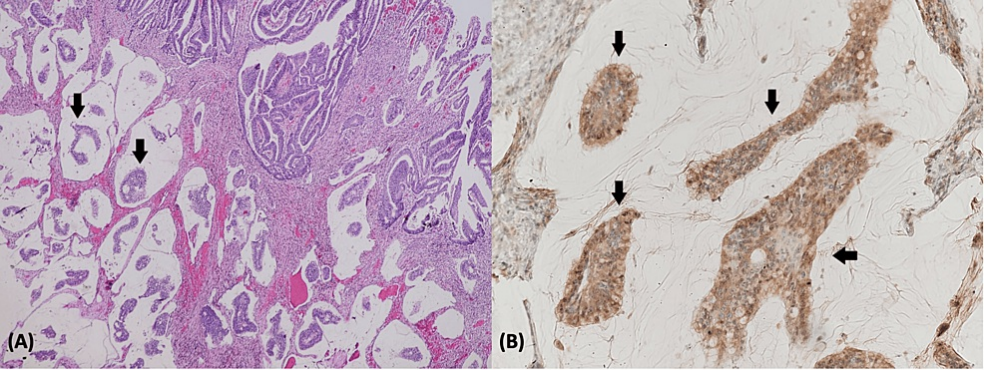
Adenocarcinoma with groups of neoplastic cells (black arrows) floating in pools of mucin: (A) H&E staining; (B) positive immunohistochemistry for AFP (x200). AFP, alpha-fetoprotein; H&E, hematoxylin and eosin

The histopathological examination revealed tumor infiltration extending through the mucosa and muscle to the serosa, with deposits also present in subserosal lymphatics. Metastasis was observed in all 10 examined lymph nodes. Immunohistochemical analysis showed that the tumor was mismatched repair protein proficient (MLH1/MSH2/MSH6/PMS 2 positive) and human epidermal growth factor receptor 2 (HER2) positive. Interestingly, it also stained positive for AFP (Figures 3A-3B). The final diagnosis indicated mucinous carcinoma of the colon, classified as Duke C (T3N2b), with a satisfactory resected margin. The postoperative period was uneventful. The patient was started on a soft diet by postoperative day 4 and successfully discharged on day 6.

A week after the surgery, both the tumor markers CEA and AFP normalized to 2.8 and 3.2 ng/mL, respectively. The positron emission tomography scan identified only mild activity at the anastomotic site, indicating post-surgical inflammation, with no evidence of distant or local metastases. The patient then completed eight cycles (four months) of adjuvant chemotherapy with capecitabine and oxaliplatin (CAPOX) regimen. A follow-up CT scan and colonoscopy, one year after adjuvant therapy, showed no evidence of local or distant recurrence.

## Discussion

This case highlighted the unexpected incidence and importance of recognizing an AFP-producing CRC. Typically, high levels of AFP raise the suspicion of HCC or yolk sac tumor (e.g., testicular teratoma). With the previous burden of hepatitis B infection and raised serum AFP level, the initial working diagnosis was to exclude a primary liver malignancy. This explained why a triphasic CT liver was performed. The CT scan incidentally revealed a proximal colonic tumor, expediting an already planned colonoscopy. 

The first case of AFP-producing CRC was described by Nakajima et al. in 1985, and there were only a handful of cases and reports published to date in the English literature [5]. According to the study by Kong et al., the primary tumor could develop throughout the entire gastrointestinal tract. It is most commonly found in the stomach (83.1%), rectum (7.9%), colon (5.1%), duodenum (1.1%), esophagus (0.6%), ileum (0.6%), and appendix (0.6%). It is also often associated with synchronous tumors such as the stomach and rectum (0.6%) and colon and rectum (0.6%) [6].

The main distinctive features of an AFP-producing tumor include a high metastasis rate and liver metastasis at the initial diagnosis (12%-25%). In the retrospective study by Kong et al., as much as 81.9% of patients developed metachronous metastasis, and the liver was the commonest site, at a rate of 53.1%. They also found that AFP-high CRC (AFP ≥ 200 ng/mL) had a higher likelihood of developing stage IV disease and liver metastasis compared to AFP-low group AFP(<200 ng/mL) [6]. Ren et al. also supported this, showing that AFP-producing CRC had poorer differentiation (50%), advanced local invasion (80%), and up to 60% of lymph node metastasis [3]. With such clinical behaviors and no standardized treatment, they are associated with more aggressive progression and poorer prognosis, and about half of the patients die within a year of therapeutic intervention [7].

Remarkably, despite a serum AFP level of 212.6 ng/mL at presentation, our patient only demonstrated regional lymph node metastasis, with no evidence of liver or distant metastasis at diagnosis and during the 12-month surveillance follow-up scan. 

Our case was classified as a stage IIIC (T3N2M0) CRC. As he presented with complete obstruction of the transverse colon, an urgent extended right hemicolectomy was performed. He was then started on adjuvant therapy with the CAPOX regimen. This is in keeping with the recommended clinical guidelines, as outlined in the European Society of Medical Oncology (ESMO), for the management of stage III AFP-negative colorectal cancer [8]. A case reported in Taiwan with AFP-producing CRC (stage IIIc) underwent curative colectomy. The case demonstrated a successful treatment outcome, with no recurrence within five months of follow-up, despite not receiving chemotherapy [9]. Alternatively, another case report by Nakamura et al. opted for neoadjuvant chemoradiotherapy followed by surgery and three months of adjuvant chemotherapy with CAPOX for stage IIIc AFP-producing CRC. The patient had no recurrence within the six months follow-up [7]. All three different regimens had comparable good outcomes and imply the current lack of uniform standardized treatment for AFP-producing CRC. 

Several guidelines such as the European Association for the Study of the Liver (EASL), the American Association for the Study of Liver Disease (AASLD), and the Asian Pacific Association for the Study of Liver Disease (APASL) discourage the use of serum AFP alone as a surveillance tool for the screening of HCC due to its low specificity and sensitivity [10,11]. This is also supported by a review that showed the role of AFP as a surveillance tool only had 60% sensitivity and 35.8% specificity for HCC [12]. Another systematic review for screening of HCC also revealed that the sensitivity of using AFP was only 41%-65% and specificity at 80%-94% [13]. Despite this, the majority in Europe, North America, and especially Asia, still routinely use it as a screening test, complementary to ultrasound use [11]. The aim is to detect HCC early as studies have shown that AFP can increase early detection from 32% to 63% [14]. 

In this case, it is crucial to emphasize that monitoring the serum AFP level carries significant implications. It serves not only to detect the recurrence of AFP-producing CRC or liver metastases but also plays a role in the early detection of HCC, given the patient’s history of hepatitis B infection. We recommend regular monitoring of AFP/CEA level every three to six months, ultrasound follow-up every six months, and annual surveillance CT scan with colonoscopy.

## Conclusions

In conclusion, the measurement of serum AFP levels may serve a dual purpose, not only in screening for HCC but also for CRC. The complexity and challenges presented in this case emphasized the importance of a multidisciplinary team approach to reach a consensual agreement for optimal management. Despite this subtype of CRC being linked with a poorer prognosis, the patient remained successfully disease-free one year after treatment. Particularly, this case report highlights the significance of recognizing atypical blood results promptly, as timely intervention plays a crucial role in preventing a potential oversight in the diagnosis of CRC. The limitation of this case is that we could have opted for a minimally invasive method, such as a laparoscopic approach, to ensure a faster recovery.
